# Attenuation of Cytotoxic Natural Product DNA Intercalating Agents by Caffeine

**DOI:** 10.3797/scipharm.1107-19

**Published:** 2011-09-17

**Authors:** Gabrielle M. Hill, Debra M. Moriarity, William N. Setzer

**Affiliations:** 1 Department of Chemistry, University of Alabama in Huntsville, Huntsville, Alabama 35899, USA; 2 Department of Biological Sciences, University of Alabama in Huntsville, Huntsville, Alabama 35899, USA

**Keywords:** Caffeine, DNA Intercalation, Cytotoxicity, π-π Complex, Density Functional Theory

## Abstract

Many anti-tumor drugs function by intercalating into DNA. The xanthine alkaloid caffeine can also intercalate into DNA as well as form π-π molecular complexes with other planar alkaloids and anti-tumor drugs. The presence of caffeine could interfere with the intercalating anti-tumor drug by forming π-π molecular complexes with the drug, thereby blocking the planar aromatic drugs from intercalating into the DNA and ultimately lowering the toxicity of the drug to the cancer cells. The cytotoxic activities of several known DNA intercalators (berberine, camptothecin, chelerythrine, doxorubicin, ellipticine, and sanguinarine) on MCF-7 breast cancer cells, both with and without caffeine present (200 μg/mL) were determined. Significant attenuation of the cytotoxicities by caffeine was found. Computational molecular modeling studies involving the intercalating anti-tumor drugs with caffeine were also carried out using density functional theory (DFT) and the recently developed M06 functional. Relatively strong π–π interaction energies between caffeine and the intercalators were found, suggesting an “interceptor” role of caffeine protecting the DNA from intercalation.

## Introduction

Caffeine is ingested by millions of people on a daily basis in tea, coffee, soft drinks, and various other foods. Because of its wide-spread usage, caffeine has been the focus of many studies for their effects on the body [1]. Overuse of caffeine is associated with heart problems, addiction due to its stimulant qualities and reproductive problems. However, it acts as a vasodilator, and hence alleviates pain associated with migraines, and moderate use of caffeine is linked to prevention of some autoimmune diseases, diabetes, and it can act as an anti-oxidant [1]. The actual interaction between caffeine and DNA is a controversial subject; it is not fully understood, but a possible scenario is that it intercalates with DNA.

Caffeine is a planar aromatic xanthine alkaloid which leads to the hypothesis that it could very easily form π–π complexes with other planar aromatic molecules such as nucleobases in DNA and several types of anticancer drugs known to intercalate DNA based on their planar structures [2, 3]. Caffeine has been shown to associate with the intercalators doxorubicin [4–6], mitoxantrone [5–7], topotecan [8], acridine orange [9], and with itself [10]. It has been previously confirmed that caffeine and theophylline can protect DNA by affecting the binding of toxic compounds [1, 11, 12]. However, in cancer patients undergoing chemotherapy, this property is not preferable. Several chemotherapy drugs such as doxorubicin and daunorubicin work by intercalating into DNA, and caffeine has been shown to reduce the toxicity of these drugs [1, 13]. Previous studies have shown that caffeine forms “stacking complexes” with these anti-cancer drugs which affects the binding of the drugs into the DNA and regulates the movement through cell membranes, and also that caffeine actually displaces drugs that have already been bound to DNA [1].

**Fig. 1:**
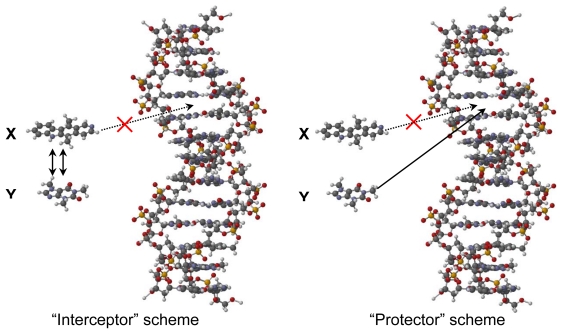
Modulation of DNA intercalation by caffeine via “interceptor” (left) or “protector” (right) interactions [15].

Two mechanisms have been proposed for the modulation of DNA intercalating drugs [14, 15], which involve equilibria of complexed drug and caffeine, caffeine and DNA, and drug and DNA. The mechanism proposed works within a system that consists of two ligands in the presence of DNA. X represents the first ligand, which is the anticancer drug, and Y represents the other binding molecule such as caffeine. The caffeine molecule (Y) can essentially do two things. It can either bind to the anticancer drug, acting as the “interceptor” molecule or it can bind to the DNA, acting as the “protector” molecule as it is “protecting” the DNA molecule from being bound by the anticancer drug (X) (Fig. 1).

In order to provide additional insight into the molecular interactions of caffeine with intercalating natural products and with DNA, we have examined: (a) the cytotoxic activities of the antitumor agents chelerythrine, camptothecin, ellipticine, doxorubicin, berberine, and sanguinarine, both alone and in the presence of caffeine, on the MCF-7 human breast adenocarcinoma cell line; (b) molecular docking of caffeine and the antitumor agents with DNA; and (c) molecular modeling of π–π interactions between caffeine and the antitumor agents and between these compounds and the guanine-cytosine base pair using density functional theory.

## Results and Discussion

### Cytotoxic Activity

*In-vitro* cytotoxicity assays on MCF-7 cells were carried out for six compounds (berberine, camptothecin, chelerythrine, doxorubicin, ellipticine, and sanguinarine) at various concentrations in order to determine their *IC*_50_ values. In a separate assay, the medium was supplemented with caffeine at a concentration of 200 μg/mL (previously determined to have little effect on the cells), and *IC*_50_ values of the compounds re-determined in the presence of caffeine. The effects of caffeine on the cytotoxic activity of the intercalating antitumor agents are shown in Table 1.

Caffeine resulted in attenuation on the cytotoxic activities of all the intercalating drugs in the present study. Some of the intercalating drugs were more affected than others as shown in their *IC*_50_ calculation in Table 1. Significant attenuation (P < 0.001) was observed for all intercalating drugs in this study; large attenuations were observed for berberine, camptothecin, doxorubicin and ellipticine, while chelerythrine and sanguinarine were marginally attenuated. The increased in *IC*_50_ due to caffeine may be attributed to either caffeine competing for intercalation sites in DNA (the “protector” scheme), or that the caffeine has formed a π–π complex with the intercalating drug (the “interceptor” scheme, Fig. 1) [15].

The slight attenuation in the *IC*_50_ values of sanguinarine and chelerythrine from the system with no caffeine to the system with caffeine could indicate there is another mechanism of cytotoxic activity in addition to DNA intercalation. It has been reported that sanguinarine induces oxidation within the cell which causes double-stranded DNA breaks [16]. Additionally, in some cell lines, sanguinarine was found to induce caspase activation [17] or severe glutathione depletion [18, 19], leading to apoptosis. It has also been suggested that both sanguinarine and chelerythrine produce H_2_O_2_ and other reactive oxygen species, which cause oxidation and subsequent apoptosis [20].

### Molecular Docking

The Molegro Virtual Docker (MVD) [21, 22] was used to carry out a molecular docking analysis of the intercalating antitumor agents as well as caffeine with DNA in order to compare docking energies. The molecular docking studies were based on structures of various intercalators with DNA that are available in the Protein Data Bank (PDB). A total of nine different DNA structures with two intercalation sites each were modeled in this docking study. The docking energies are summarized in Table 2.

Caffeine is the worst binding ligand according to the docking studies and averaged 4 kcal/mol weaker binding than the worst intercalating drug (ellipticine). The ligand with the strongest docking energy was doxorubicin, which docked, on average, 9.3 kcal/mol more strongly than caffeine. These docking data would suggest that displacement of intercalating antitumor agents is not a thermodynamically favorable process. Previous spectroscopic [23, 24] and theoretical [25] studies have suggested that intercalation is not the predominant mechanism for interaction of caffeine with DNA. Likewise, de-intercalation of ethidium bromide from DNA by caffeine has been attributed to caffeine–ethidium bromide stacking aggregation and not intercalation of caffeine into DNA [26].

### Density Functional Theory Calculations

In order to probe π–π complexation between caffeine and the intercalators, DFT modeling studies were carried out using Spartan ’08 for Windows [27] with the M06 functional [28] and the 6-31G* basis set. The recently developed M06 suite of density functionals [28] has been shown to give generally superior performance for non-covalent interactions such as hydrogen bonding, dipole-dipole, and π–π stacking interactions, unlike widely used B3LYP and BLYP methods [29–32]. In this work we have chosen to use the M06 hybrid functional because of its previously reported performance with π–π stacking interactions [28]. A number of different orientations were constructed and full geometry optimization was carried out. The π–π interaction energies for the lowest-energy orientations of the six intercalating compounds with caffeine are summarized in Table 3.

The M06 calculations all indicate relatively strong π–π interactions between caffeine and the antitumor drugs with gas-phase exothermic interaction energies ranging from −16.8 to −21.6 kcal/mol and aqueous energies of −11.0 to −16.5 kcal/mol. Interactions that likely contribute to these favorable π–π complexes include dipole-dipole interactions [33, 34], electrostatic interactions [35, 36] and van der Waals interactions [37], as well as frontier molecular orbital interactions [38, 39]. Frontier molecular orbital theory [40] suggests that the important interactions of caffeine with the intercalating drugs will be the HOMO of caffeine and the LUMO of the antitumor agents.

**Fig. 2:**
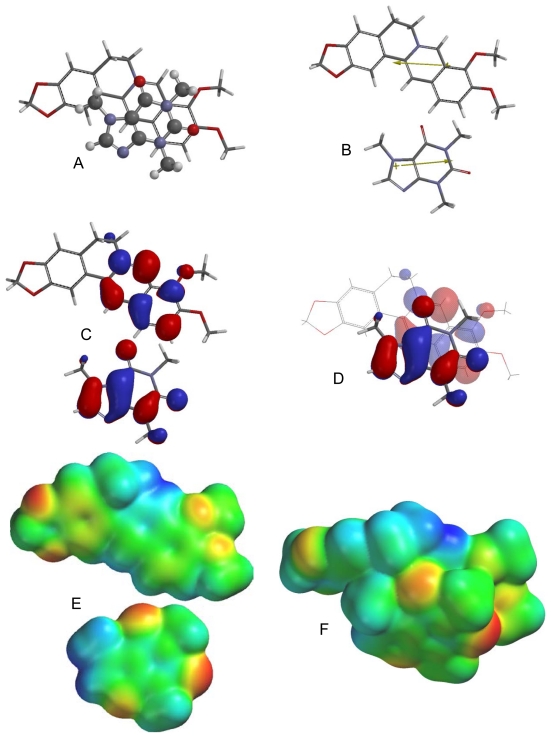
Lowest-energy orientation of the π–π complex between berberine and caffeine. (A) Face-to face orientation of caffeine (ball and spoke model) in its lowest-energy orientation with berberine (tube model). (B) Molecular dipoles of berberine (top) and caffeine (bottom). (C) LUMO of berberine (top) and HOMO of caffeine (bottom). (D) Frontier molecular orbital overlap of caffeine with berberine in the lowest-energy orientation. (E) Electrostatic potential maps of berberine (top) and caffeine (bottom). (F) Electrostatic potential map of the lowest-energy π–π complex between berberine and caffeine.

**Fig. 3:**
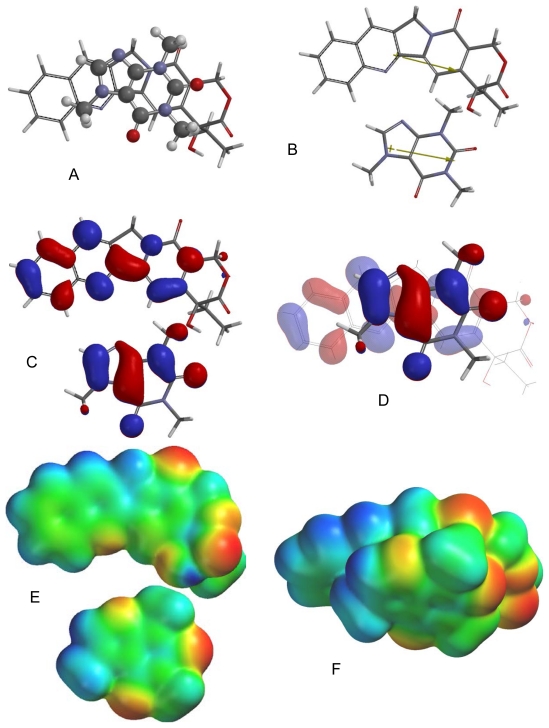
Lowest-energy orientation of the π–π complex between camptothecin and caffeine. (A) Face-to face orientation of caffeine (ball and spoke model) in its lowest-energy orientation with camptothecin (tube model). (B) Molecular dipoles of camptothecin (top) and caffeine (bottom). (C) LUMO of camptothecin (top) and HOMO of caffeine (bottom). (D) Frontier molecular orbital overlap of caffeine with camptothecin in the lowest-energy orientation. (E) Electrostatic potential maps of camptothecin (top) and caffeine (bottom). (F) Electrostatic potential map of the lowest-energy π–π complex between camptothecin and caffeine.

**Fig. 4:**
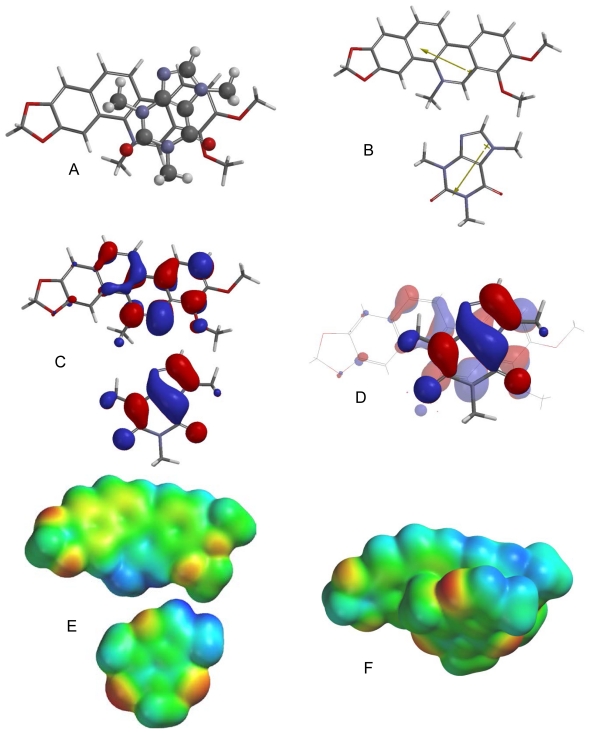
Lowest-energy orientation of the π–π complex between chelerythrine and caffeine. (A) Face-to face orientation of caffeine (ball and spoke model) in its lowest-energy orientation with chelerythrine (tube model). (B) Molecular dipoles of chelerythrine (top) and caffeine (bottom). (C) LUMO of chelerythrine (top) and HOMO of caffeine (bottom). (D) Frontier molecular orbital overlap of caffeine with chelerythrine in the lowest-energy orientation. (E) Electrostatic potential maps of chelerythrine (top) and caffeine (bottom). (F) Electrostatic potential map of the lowest-energy π–π complex between chelerythrine and caffeine.

**Fig. 5:**
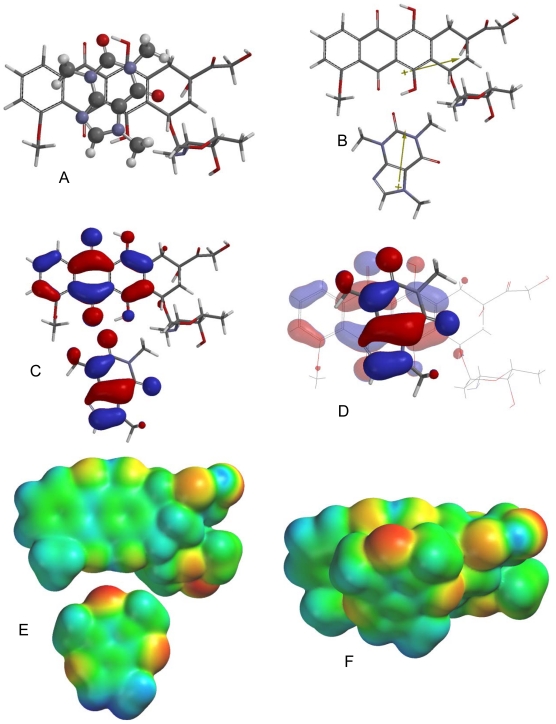
Lowest-energy orientation of the π–π complex between doxorubicin and caffeine. (A) Face-to face orientation of caffeine (ball and spoke model) in its lowest-energy orientation with doxorubicin (tube model). (B) Molecular dipoles of doxorubicin (top) and caffeine (bottom). (C) LUMO of doxorubicin (top) and HOMO of caffeine (bottom). (D) Frontier molecular orbital overlap of caffeine with doxorubicin in the lowest-energy orientation. (E) Electrostatic potential maps of doxorubicin (top) and caffeine (bottom). (F) Electrostatic potential map of the lowest-energy π–π complex between doxorubicin and caffeine.

**Fig. 6:**
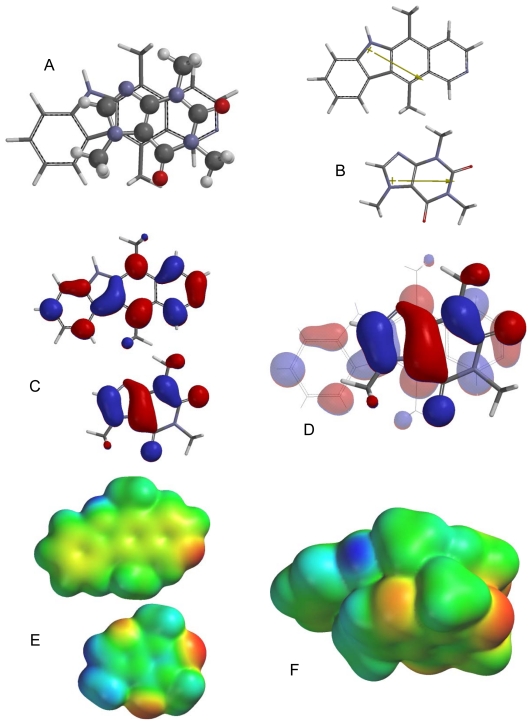
Lowest-energy orientation of the π–π complex between ellipticine and caffeine. (A) Face-to face orientation of caffeine (ball and spoke model) in its lowest-energy orientation with ellipticine (tube model). (B) Molecular dipoles of ellipticine (top) and caffeine (bottom). (C) LUMO of ellipticine (top) and HOMO of caffeine (bottom). (D) Frontier molecular orbital overlap of caffeine with ellipticine in the lowest-energy orientation. (E) Electrostatic potential maps of ellipticine (top) and caffeine (bottom). (F) Electrostatic potential map of the lowest-energy π–π complex between ellipticine and caffeine.

**Fig. 7:**
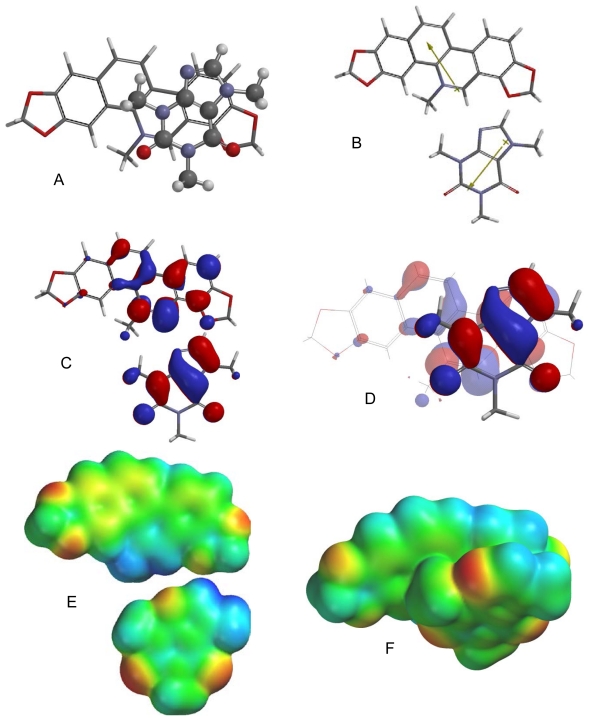
Lowest-energy orientation of the π–π complex between sanguinarine and caffeine. (A) Face-to face orientation of caffeine (ball and spoke model) in its lowest-energy orientation with sanguinarine (tube model). (B) Molecular dipoles of sanguinarine (top) and caffeine (bottom). (C) LUMO of sanguinarine (top) and HOMO of caffeine (bottom). (D) Frontier molecular orbital overlap of caffeine with sanguinarine in the lowest-energy orientation. (E) Electrostatic potential maps of sanguinarine (top) and caffeine (bottom). (F) Electrostatic potential map of the lowest-energy π–π complex between sanguinarine and caffeine.

Of the different caffeine-berberine orientations, the lowest energy orientation (Fig. 2) is such that there are favorable dipole-dipole and electrostatic interactions. Frontier molecular orbital overlap, however, is not favorable. The lowest-energy π–π orientation for caffeine with camptothecin (Fig. 3) has the molecular dipoles of the caffeine and camptothecin aligned rather than opposed and the electrostatic interactions are also unfavorable. They do, however, have favorable frontier molecular orbital alignments. In the lowest-energy π–π complex between chelerythrine and caffeine the molecular dipoles are perpendicular, frontier molecular orbital overlap is not evident, but electrostatic interactions are generally favorable (Fig. 4). The molecular dipoles for caffeine and doxorubicin are nearly perpendicular in the lowest energy orientation (Fig. 5), but there do seem to be favorable electrostatic and frontier molecular orbital interactions. In the ellipticine-caffeine complex (Fig. 6), the dipole moments unfavorably aligned, but HOMO-LUMO interactions as well as electrostatic interactions are favorable for this orientation. Sanguinarine, structurally very similar to chelerythrine, shares the same lowest-energy orientation (Fig. 7) with the same interactions: perpendicular molecular dipole moments, poor FMO overlap, but good electrostatic interactions.

The π–π interactions between caffeine and the guanine-cytosine (G-C) base pair as well as the interactions between the intercalating drugs and the G-C base pair have been modeled using DFT at the M06/6-31G* level. The starting orientations for each calculation were the two orientations of the intercalator with G-C from the X-ray crystal structure (in the case of doxorubicin [42]) or the lowest-energy docked poses from the molecular docking analyses (see above). The π–π interaction energies for the intercalators and G-C are summarized in Table 4. For this discussion, an “intercalation energy” can be defined as the average of the π–π interaction energies for each complex between intercalator and G-C. The DFT calculated “intercalation energies” closely mirror the molecular docking energies above. That is, doxorubicin is calculated to release the most amount of energy upon intercalation, while caffeine is expected to release the least. Ellipticine is also predicted to be a weaker intercalator than the other drugs, consistent with the molecular docking results.

Based on the data in this study and on previous studies [23–25], we conclude that caffeine modulates the activities of intercalating cytotoxic drugs by π–π interactions (“interceptor”) rather than intercalation of caffeine directly into DNA (“protector”) and that either frontier molecular orbital interactions, dipole-dipole interactions, and/or electrostatic interactions play important roles in the π–π orientations of caffeine with the intercalators.

## Experimental

### Cytotoxicity Assay

Human MCF-7 breast adenocarcinoma cells (ATCC No. HTB-22) [43] were grown in a 5% CO_2_ environment at 37°C in RPMI-1640 medium, supplemented with 10% fetal bovine serum, 100,000 units penicillin and 10.0 mg streptomycin per liter of medium, 15 mM of Hepes, and buffered with 26.7 mM NaHCO_3_, pH 7.35.

MCF-7 cells were plated into 96-well cell culture plates at 1.0 × 10^4^ cells per well, with the volume in each well of 100 μL. After 48 h, the supernatant fluid was removed by suction and replaced with 100 μL growth medium containing test compounds at different concentrations; with or without caffeine (200 μg/mL). Solutions were added to wells in eight replicates. Medium alone was used as a negative control and tingenone (100 μg/mL) was used as a positive control [44]. After the addition of compounds, plates were incubated for 48 hr at 37°C; medium was then removed by suction and the MTT assay for cell viability was carried out [45]. The plates were then incubated for fifteen minutes and colorimetric readings were recorded (using a Molecular Devices SpectraMAX Plus 384 microplate reader, 570 nm). Average absorbances, standard deviations, and percent kill ratios (%kill_cmpd_/%kill_control_) were calculated.

### Molecular Docking

Molecular structures for the compounds were built using Spartan ’08 for Windows [27], and geometries optimized using the MMFF 94 force field [46]. Docking studies of caffeine and the intercalating drugs were carried out based on the structures of DNA complexed with *bis*-daunorubicin (PDB: 1AL9) [47], doxorubicin (PDB: 1P20) [42], ellipticine (PDB: 1Z3F) [48], cryptolepine (PDB: 1K9G) [49], 9-amino-*N*-[2-(4-morpholinyl)ethyl]-4-acridinecarboxamide (PDB: 1KCI) [50], 5-fluoro-9-amino-(*N*-(2-dimethylamino)ethyl)-acridine-4-carboxamide (PDB: 1DL8) [51], 5-bromo-9-amino-(*N*-(2-dimethylamino)ethyl)-acridine-4-carboxamide (PDB: 367D) [52], 9-amino-(*N*-(2-dimethylamino)ethyl)acridine-4-carboxamide (PDB: 452D [52] and 465D [53]). The structures were downloaded from the Protein Data Bank (PDB) using Molegro Virtual Docker v. 4.3 [21]. These PDB structures provide a selection of DNA structures with known planar aromatic intercalators. All solvent molecules and the co-crystallized inhibitor were removed from the structures to provide sterically unimpeded cavities for ligand docking. Molecular docking calculations for the ligands at the intercalation sites of DNA were undertaken using the MolDock docking algorithm of MVD v. 4.3 [22]. A sphere with a 10 Å radius (large enough to completely encompass the cavity/intercalation site) was centered on the intercalation site in order to allow each ligand to explore potential binding poses. The lowest-energy docking poses are summarized in Table 2.

### Density Functional Molecular Structures and Energies

The calculations were carried out using SPARTAN ’08 for Windows [27]. The recently developed M06 [28] functional was used together with the 6-31G* basis set [54] for the optimization of all stationary points in the gas phase. Several different orientations of the drugs (berberine, camptothecin, chelerythrine, doxorubicin, ellipticine, and sanguinarine) and caffeine (3.4 Ǻ separation) were carried out with complete geometry optimization. Interaction energies using the SM5.4 aqueous solvation model [41] were determined using the gas-phase geometries. Interactions between intercalators and G-C were carried out starting with the two orientations of the intercalator with G-C from the X-ray crystal structure in the case of doxorubicin [42], or the lowest-energy docked poses from the molecular docking analyses (see above) and starting with a π–π separation of 3.4 Ǻ. The geometries of each complex were completely optimized at the M06/6-31G* level of theory.

## Figures and Tables

**Tab. 1:** Cytotoxicity attenuation of intercalating antitumor agents by caffeine.

Compound	*IC*_50_ (μM)^a^	
	no caffeine	with caffeine^b^
Berberine	50.0(4.3)	511(59)
Camptothecin	9.21(1.41)	335(31)
Chelerythrine	32.1(0.5)	37.4(0.8)
Doxorubicin	11.4(2.2)	31.7(3.9)
Ellipticine	12.8(0.5)	333(7)
Sanguinarine	5.78(0.22)	6.66(0.16)

a Standard deviations in parentheses.

b Caffeine concentration = 1030 μM.

**Tab. 2:** Molegro Virtual Docker (MVD) docking energies (kcal/mol) of caffeine and intercalating antitumor agents with DNA.

Ligand	1AL9^a^		1K9G		1KCI		1P20		1Z3F	

	1^b^	2^b^	1	2	1	2	1	2	1	2
Caffeine	−14.9	−15.0	−16.5	−16.4	−16.3	−16.5	−16.0	−16.0	−16.4	−16.5
Berberine	−18.5	−18.8	−22.9	−22.3	−23.2	−22.7	−17.7	−18.1	−20.0	−21.6
Camptothecin	−18.5	−18.6	−22.0	−22.0	−22.2	−22.6	−19.4	−19.3	−22.1	−22.4
Chelerythrine	−19.7	−20.4	−21.1	−21.4	−22.8	−22.2	−20.2	−20.2	−21.6	−21.9
Doxorubicin	−26.7	−26.0	−25.3	−25.3	−26.1	−26.1	−25.7	−25.3	−23.7	−23.5
Ellipticine	−18.1	−17.8	−20.8	−20.8	−20.8	−20.8	−18.8	−18.6	−21.3	−21.4
Sanguinarine	−20.1	−20.1	−21.6	−21.5	−22.7	−23.2	−20.3	−20.1	−22.1	−22.5

**Ligand**	**1DL8**		**367D**		**452D**		**465D**		**Ave**^c^	

	**1**	**2**	**1**	**2**	**1**	**2**	**1**	**2**		

Caffeine	−16.4	−16.5	−16.4	−16.7	−16.5	−16.7	−16.5	−16.7	−16.3	
Berberine	−23.1	−23.3	−22.9	−20.1	−23.2	−23.4	−19.1	−20.1	−21.2	
Camptothecin	−22.2	−22.6	−22.4	−22.8	−22.1	−23.3	−21.6	−22.9	−21.6	
Chelerythrine	−22.6	−21.6	−22.9	−22.4	−23.1	−23.1	−21.9	−22.6	−21.8	
Doxorubicin	−25.7	−25.3	−25.4	−27.3	−26.5	−26.7	−23.9	−26.4	−25.6	
Ellipticine	−20.9	−21.0	−21.0	−21.0	−20.7	−20.9	−20.5	−20.5	−20.3	
Sanguinarine	−22.4	−22.9	−22.7	−23.3	−23.0	−23.8	−21.8	−23.3	−22.1	

a Protein Data Bank identification number.

b Intercalation site number in the DNA structure.

c Average intercalation docking energy for all structures.

**Tab. 3:** π–π Interaction energies of intercalating antitumor agents and caffeine.

Compounds	*E*_vac_ (kcal/mol)^a^	*E*_aq_ (kcal/mol)^b^
Caffeine + Berberine	−19.34	−12.60
Caffeine + Camptothecin	−18.04	−13.05
Caffeine + Chelerythrine	−20.54	−14.12
Caffeine + Doxorubicin	−19.16	−15.86
Caffeine + Ellipticine	−16.83	−11.02
Caffeine + Sanguinarine	−21.60	−16.47

a Calculated interaction energies in the gas phase.

b Calculated interaction energies using an aqueous solvation model (SM5.4 [41]).

**Tab. 4:** π–π Interaction energies for intercalators with guanine-cytosine base pair.

Compounds	*E* (kcal/mol)		
	Orientation 1	Orientation 2	Average
Caffeine	−17.3	−22.1	−19.7
Berberine	−30.9	−29.4	−30.2
Camptothecin	−25.4	−22.3	−23.9
Chelerythrine	−25.5	−30.6	−28.0
Doxorubicin	−29.1	−31.6	−30.3
Ellipticine	−20.3	−20.8	−20.5
Sanguinarine	−28.3	−30.3	−29.3
